# The President’s Wound

**Published:** 1901-11

**Authors:** J. S. Poynor

**Affiliations:** Bartlett, Texas


### Correspondence.

### The President's Wound.

BARTLETT, TEXAS, October 8, 1901.

*Editor Texas Medical Journal.*

I have just read yours on the McKinley wound. I don't think you exactly cover the ground. Bullet wounds, until the last year or two, were properly classed as "contused wounds." The vast majority of the wounds you saw during the war sloughed around the track of the bullet. The tissues were contused to such a degree as to be devitalized to the extent of a quarter to a half of an inch or even more. This devitalized tissue had to slough off, and this is the gangrene that made Mr. McKinley's wound fatal. Simply stitching up the holes in the stomach, the sutures in dead tissue, prevented effusion of contents of stomach for a while—till the slough came away; then he was practically bound to die, without a new operation.

Slow moving bullets from old style arms and little short bulldog pistols, make contused wounds, and as a rule they slough all around the track of the bullet. Small balls from the Mauser and Kraig-Jorgensen, etc., with exceedingly high velocity, make practically incised wounds. A boy breaks a window pane all to pieces with a rock. A rifle ball makes a clean hole through it.

On these grounds I predicted the President's death.

You saw thousands of bullet wounds during the war. So did I. I saw a few heal without sloughing. These were from rifles at close quarters, where the initial velocity was very great, I think. One of these short pistols cannot impart sufficient velocity to a ball to make an incised wound.

J. S. POYNOR, M. D.

